# Telerehabilitation Delivery in Canada and the Netherlands: Results of a Survey Study

**DOI:** 10.2196/45448

**Published:** 2023-02-20

**Authors:** Edward Giesbrecht, Mel E Major, Moni Fricke, Pamela Wener, Maarten van Egmond, Jesse J Aarden, Cara L Brown, Margriet Pol, Marike van der Schaaf

**Affiliations:** 1 Department of Occupational Therapy College of Rehabilitation Sciences University of Manitoba Winnipeg, MB Canada; 2 Department of Physical Therapy Faculty of Health Amsterdam University of Applied Sciences Amsterdam the Netherlands; 3 Research Group Occupational Therapy: Participation and Environment Center of Expertise Urban Vitality, Faculty of Health Amsterdam University of Applied Sciences Amsterdam the Netherlands; 4 Amsterdam Movement Sciences Ageing and Vitality Amsterdam the Netherlands; 5 Department of Physical Therapy College of Rehabilitation Sciences University of Manitoba Winnipeg, MB Canada; 6 European School of Physiotherapy Faculty of Health Amsterdam University of Applied Sciences Amsterdam the Netherlands; 7 Department of Medicine for Older People Amsterdam UMC location Vrije Universiteit Amsterdam Amsterdam the Netherlands; 8 Amsterdam Public Health Aging & Later Life Amsterdam the Netherlands; 9 Rehabilitation Medicine, Meibergdreef 9 Amsterdam UMC location University of Amsterdam Amsterdam the Netherlands

**Keywords:** telerehabilitation, digital health, telehealth, eHealth, competencies, capabilities, mobile phone

## Abstract

**Background:**

Following the onset of the COVID-19 pandemic, telerehabilitation (TR) has been expanding to address the challenges and risks of in-person delivery. It is likely that a level of TR delivery will continue after the pandemic because of its advantages, such as reducing geographical barriers to service. Many pandemic-related TR initiatives were put in place quickly. Therefore, we have little understanding of current TR delivery, barriers and facilitators, and how therapists anticipate integrating TR into current practice. Knowing this information will allow the incorporation of competencies specifically related to the use and provision of TR into professional profiles and entry-to-practice education, thereby promoting high-quality TR care.

**Objective:**

This study aimed to obtain a descriptive overview of current TR practice among rehabilitation therapists in Canada and the Netherlands and identify perceived barriers to and facilitators of practice.

**Methods:**

A web-based cross-sectional survey was conducted with occupational, physical, and respiratory therapists and dietitians in Canada (in French and English) and the Netherlands (in Dutch and English) between November 2021 and March 2022. Recruitment was conducted through advertisements on social media platforms and email invitations facilitated by regulatory and professional bodies. The survey included demographic and practice setting information; whether respondents delivered TR, and if so, components of delivery; confidence and satisfaction ratings with delivery; and barriers to and facilitators of use. TR satisfaction and uptake were measured using the Telehealth Usability Questionnaire and modified Technology Acceptance Model. Data were first summarized descriptively, and then, comparisons were conducted between professions.

**Results:**

Overall, 723 survey responses were received, mostly from Canada (n=666, 92.1%) and occupational therapists (n=434, 60%). Only 28.1% (203/723) reported receiving specific training in TR, with 1.2% (9/723) indicating that it was part of their professional education. Approximately 19.5% (139/712) reported not using TR at all, whereas most participants (366/712, 51.4%) had been using this approach for 1 to 2 years. Services delivered were primarily teleconsultation and teletreatment with individuals. Respondents offering TR were moderately satisfied with their service delivery and found it to be effective; 90.1% (498/553) indicated that they were likely to continue offering TR after the pandemic. Technology access, confidence, and setup were rated the highest as facilitators, whereas technology issues and the clinical need for physical contact were the most common barriers.

**Conclusions:**

Professional practice and experience with TR were similar in both countries, suggesting the potential for common strategic approaches. The high prevalence of current practice and strong indicators of TR uptake suggest that therapists are likely to continue TR delivery after the pandemic; however, most therapists (461/712, 64.7%) felt ill prepared for practice, and the need to target TR competencies during professional and postprofessional education is critical. Future studies should explore best practice for preparatory and continuing education.

## Introduction

### Background

The use of digital technologies in the health care sector is developing rapidly. The term, *eHealth*, is an umbrella term for combining technology and health, defined by the World Health Organization as “the cost-effective and secure use of information and communications technologies (ICT) in support of health and health-related fields, including healthcare services, health surveillance, health literature, and health education, knowledge and research” [1]. Recently, *digital health* was described as a term “encompassing eHealth, as well as emerging areas, such as the advanced computing sciences in ‘big data,’ genomics and artificial intelligence” [2]. Digital *interventions* are further defined as “a discrete functionality of digital technology that is applied to achieve health objectives” [2]. Within this broad field of digital health, *telehealth*, *telemedicine*, and *telerehabilitation* (TR) are often used interchangeably [3]. Telehealth encompasses the use of information and communications technology (ICT) for “the application of evaluative, consultative, preventative, and therapeutic services” [4], whereas telemedicine applies to the use of ICT for the delivery of direct clinical services and *TR* refers to the digital delivery of rehabilitation services [5,6].

### TR and the COVID-19 Pandemic

With the increasing advancement and availability of ICT, TR has become more attractive to health care professionals, service recipients, and insurance companies. Although TR was becoming more common before the COVID-19 pandemic, occupational therapists (OTs), physical therapists (PTs), and respiratory therapists (RTs) were compelled to quickly adopt these alternative strategies to address access, efficiency, and effectiveness in clinical service provision during the COVID-19 pandemic [7,8]. However, barriers to broad TR adoption and access persist. Accessibility is affected by factors at the *service provider* level, such as comfort or competence with eHealth delivery or the availability and systemic support of eHealth apps, or at the *service recipient* level, such as access to technology and internet and the applicability of eHealth apps for users with impaired health, digital literacy, or variations in cultural backgrounds [9-11].

Therapists have turned to TR as a strategy to maintain continuity of care and access to treatment during the COVID-19 pandemic [12]. TR delivery can include web-based coaching sessions (either group or individual), by using existing eHealth apps and wearables such as activity trackers, through telephone or video consultations, and by sharing educational material through the web (such as instructive videos on YouTube) [13,14]. In the Canadian and Dutch contexts, we have limited information about how therapists have chosen to implement eHealth services as part of rehabilitation interventions. As we approach a point where COVID-19 conditions stabilize, we are uncertain about which of these new or alternative ways of providing interventions will remain as *current practice* moving forward. However, given that TR was already gaining momentum in both countries before the pandemic, it is a reasonable assumption that it will be applied more frequently in daily clinical practice.

Importantly, many TR initiatives imposed owing to COVID-19 conditions were expedient, without adequate preparation of the provider or recipient of service [15,16]. TR is likely to continue after the pandemic, because of some of the advantages it affords, and thus, it is increasingly important that therapists entering practice are equipped with the necessary eHealth competencies. Currently, newly graduated rehabilitation professionals have limited exposure to, or experience with, the delivery of digital interventions, let alone competence to assess the efficacy of such interventions [9,15,16]. Some studies have started trying to identify the competencies required for TR delivery to help guide educational programs and professional continuing education. Davies et al [17] recently released a capability framework for quality care videoconferencing delivered by PTs, which includes 7 domains: compliance, patient privacy and confidentiality, patient safety, technology skills, telehealth delivery, assessment and diagnosis, and care planning and management. However, without knowing the current state of TR delivery, it is difficult to know how to apply these competencies or whether they address the knowledge needs of different types of rehabilitation therapists currently delivering these services.

### Context of Practice

Between Canada and the Netherlands, a similar need for exploration and further development of TR services can be identified, albeit for different reasons. In Canada, TR may deliver health care services to rural and remote areas, creating solutions for patients who are otherwise not able to receive face-to-face services at hospitals or clinics [18]. In the Netherlands and Canada, TR services may help to deliver health care services to the growing number of people with complex health care needs in the context of increasing shortages of health care professionals and health care funding [19,20]. Although there are fundamental differences between the Canadian and Dutch health care systems, many similarities can be identified. Both countries offer universal health care access; however, in Canada, a single government-run scheme is funded through taxation, whereas the Netherlands uses mandatory private insurance plans and predominantly private hospitals. Both countries emphasize building a strong primary care system through primary care renewal [21,22]. In both Canada and the Netherlands, access to rehabilitation is being addressed by the inclusion of technology. However, a substantial proportion of PTs in both countries work in a fee-for-service model, in which care recipients must either pay out of pocket or arrange third-party coverage; this is particularly true for neuromusculoskeletal conditions. Another similarity has been the increased emphasis on population health, with increased rehabilitation services targeting health promotion and disease prevention [23].

Given these similarities in practice and health priorities, a collaborative research group with investigators at the University of Manitoba and the Amsterdam University of Applied Sciences explored current (peri–COVID-19) TR practice in the Canadian and Dutch contexts and therapists’ perceptions of barriers to and facilitators of TR practice. We were specifically interested in documenting whether therapists were using TR in daily practice and for what purposes, which types of platforms and services were used, barriers and facilitators associated with these services, perceptions of preparation for and current delivery of TR, and uptake and intent for future TR delivery. If such information exists, appropriate evaluations of service delivery and strategic planning for rehabilitation service delivery after the pandemic can be performed. Therefore, this study aimed to obtain a descriptive overview of current TR practice among rehabilitation professionals in Canada and the Netherlands and identify perceived barriers to and facilitators of practice.

## Methods

### Design

We administered a web-based survey, using the SurveyMonkey platform (Momentive), to gather participants’ experiences with TR practice. The survey method was the most efficient and accessible approach to access various disciplines across wide geographical regions and in multiple languages (ie, English, French, and Dutch). The survey questions addressed demographics, description of current practice, identification of facilitators and barriers, and rating of several TR use metrics and included validated measures of TR usability and uptake.

### Participants

We specifically targeted rehabilitation therapists from the professional programs in our universities. In Canada, this included OTs, PTs, and RTs, and in the Netherlands, this included OTs, PTs, exercise therapists (ETs), and dietitians (DTs). Participation was restricted to therapists with a minimum of 6 months of work experience at the time of the survey but was open to those who had not used TR in their practice.

### Recruitment

Recruitment in Canada followed 2 main strategies. First, provincial regulatory and professional organizations for OTs, PTs, and RTs were contacted with a request to distribute survey invitations to their registrants or members using their email distribution lists. Both French-language and English-language invitations were made available. For organizations that agreed, introductory emails were distributed, followed by subsequent reminder emails at 2 and 4 weeks. Second, invitations to participate were posted on a variety of social media pages including those of all 3 national professional associations and several provincial regulatory or professional bodies and via social media accounts of the research team (eg, Twitter, Facebook, Instagram, and LinkedIn).

Recruitment in the Netherlands was conducted via social media posts (eg, LinkedIn and Facebook) and through direct email invitations sent to lecturing staff at the university PT, OT, DT, and ET programs and therapists participating in the Rehabilitation After Critical Illness and Hospital Discharge interprofessional primary care network [24]. In addition, a web page was designed and placed on the website of the Amsterdam University Medical Center and the Amsterdam University of Applied Sciences expertise center, *Nutrition and Exercise Now* [25]. The recruitment strategies used in both Canada and the Netherlands invited participation from therapists working in any context (ie, age or diagnostic group and private or public funding).

### Survey Development

The survey tool was developed by the research team and included members with expertise in TR practice and survey development and implementation. Survey development was informed by the Association for Medical Education in Europe Guidelines for educational research [26] and a review of the literature, including previously published TR surveys. Particular attention was given to the quality of the survey questions, avoiding common pitfalls such as agreement response items, unevenly spaced and unlabeled response options, and multibarreled questions [27]. Although all questions were structured to select ≥1 options, some also provided open text space for comments to further elaborate or explain. A draft version was pilot-tested by a rehabilitation graduate student, resulting in several content and formatting improvements. The first section included questions about demographics, training, and clinical practice and ended with a question about current TR delivery. The second section, provided only to those delivering TR, asked about the type of TR offered, how this was provided, experiences with TR delivery including facilitators and barriers, and usability of TR. The final section, provided to all respondents, inquired about TR acceptability and uptake.

Overall, 4 self-rating questions, using 5-point Likert scales, were developed to assess *experience* and *confidence* in providing TR (for all respondents) and perceived *effectiveness* and *satisfaction* with TR delivery (for respondents who had used TR). We also incorporated 2 standardized and validated measures: the Telehealth Usability Questionnaire (TUQ) [28] and the modified Technology Acceptance Model (mTAM) [29]. The TUQ is composed of 21 statements regarding the usability of TR, each with 7 response options ranging from completely disagree to completely agree; this was provided only to those respondents who had used TR. The mTAM assesses factors related to acceptance and uptake of TR as a clinical tool and was included for all respondents. It is composed of 33 statements with 7 response options regarding agreement; 1 item was removed because it was not relevant to the target population.

The final survey was translated into French using key elements for evidence-informed translation [30]. The translation was conducted by a research assistant fluent in French and English and then blindly back-translated by a bilingual coinvestigator. Both documents were reviewed by a fully bilingual third party to verify the accuracy for French grammar and cultural relevance. After piloting this version, minor wording changes were made to improve clarity. Next, the survey was translated into Dutch by a research assistant who is a native speaker and fluent in English. The translated version and the original survey were carefully reviewed by bilingual members of the research team. The survey was administered using the SurveyMonkey web-based platform with an anonymous response option (excluding email address and IP address) to ensure anonymity. Potential participants were provided with a direct link to the survey via the invitation email. Data were collected between November 2021 and March 2022.

### Analysis

Data from each survey were exported directly from the SurveyMonkey platform into Microsoft Excel (Microsoft Corp) spreadsheets and then consolidated in a single document. Qualitative (open text) responses were then extracted into a separate spreadsheet with corresponding respondent ID numbers, where they could be sorted. Analysis was conducted using Microsoft Excel (version 16.54) and SPSS (version 27; IBM Corp). Survey responses were reported with summary statistics, using frequency and distribution (mean, SD, and percentage). Group comparison of continuous data was conducted using independent samples *t* test (2-tailed) or ANOVA (with adjustment when equal variance could not be assumed). For comparisons with categorical data, we used chi-square tests.

There was response attrition in some surveys, resulting in some partially complete data sets. The available responses for each survey question were included in descriptive statistics (with the appropriate n indicated), and pair-wise deletion was used for variable comparisons. In most cases, the small number of responses among DTs and RTs resulted in their exclusion from comparative analyses.

Open-ended responses were analyzed in 2 different ways, depending on the nature of the open-ended question. For questions to which the open-ended response was the “other” option, we incorporated responses back into the close-ended response options where appropriate. The remaining responses were categorized by one researcher (JA) and reviewed by a second researcher (CB). Each individual open-ended response potentially contained multiple content topics. Thus, each response was broken down into these individual topics, and similar topics were grouped together to form a coding framework. Once the initial coding framework was completed, the number of responses in each code was counted, and codes with very few responses were examined to determine whether there were similar ideas that could be combined. This process was continued until the codes were developed into categories that were representative of the results. Any discrepancies between the 2 researchers were resolved through discussion.

### Ethics Approval

All participants confirmed that they were providing informed consent at the beginning of the survey questionnaire before proceeding to the questions, in accordance with the regulations at both universities. Ethics approval was obtained from the University of Manitoba human research ethics board (HS25158[H2021:330]) in Canada and the Amsterdam University of Applied Science research ethics committee (2021-131350) in the Netherlands.

## Results

### Participant Demographics

We received a total of 723 usable survey responses (ie, those responding to at least one question), with 666 (92.1%) from Canada and most (n=434, 60%) from OTs; only 6 (0.8%) responses were from DTs, and no ETs responded. Complete responses (ie, all questions are answered) were available for 83.8% (606/723) of the surveys. Respondents predominantly had >10 years of clinical experience; approximately half of the respondents (321/723, 44.4%) reported private practice being at least part of their practice, and most respondents (597/723, 82.6%) worked with the adult population. Table 1 shows respondents’ characteristics.

**Table 1:** Respondent characteristics with number of responses.

			Total respondents (N=723), n (%)		Site, n (%)					Profession, n (%)						
					Canadian (n=666, 92.1%)		Dutch (n=57, 7.8%)		OT^a^ (n=434, 60%)			PT^b^ (n=233, 32.2%)		RT^c^ (n=50, 6.9%)		DT^d^ (n=6, 0.8%)
Complete data			606 (83.8)		565 (84.8)		41 (71.9)		375 (86.4)			190 (81.5)		36 (72)		5 (83.3)
**Time in practice (years)**																
	0-3	77 (10.7)		66 (9.9)		11 (19.3)		43 (9.9)			25 (10.7)		5 (10)		4 (66.7)	
	3-5	59 (8.2)		55 (8.3)		4 (7)		45 (10.4)			12 (5.2)		2 (4)		0 (0)	
	5-10	95 (13.1)		86 (12.9)		9 (15.8)		61 (14.1)			28 (12)		5 (10)		1 (16.7)	
	>10	492 (68)		459 (68.9)		33 (57.9)		285 (65.7)			168 (72.1)		38 (76)		1 (16.7)	
**Practice location^e^**																
	Private practice	321 (44.4)		290 (43.5)		31 (54.4)		189 (43.5)			125 (53.6)		5 (10)		2 (33.3)	
	Hospital	164 (22.7)		154 (23.1)		10 (17.5)		66 (15.2)			61 (26.2)		34 (68)		3 (50)	
	Rehabilitation center	116 (16)		104 (15.6)		12 (21)		91 (20.9)			20 (8.6)		3 (6)		2 (33.3)	
	Community	96 (13.3)		96 (14.4)		0 (0)		67 (15.4)			29 (12.4)		0 (0)		0 (0)	
	Education system	35 (4.9)		29 (4.4)		6 (10.5)		22 (5.1)			12 (5.2)		1 (2)		0 (0)	
	Long-term care	26 (3.6)		18 (2.7)		8 (14)		21 (4.8)			3 (1.3)		1 (2)		1 (16.7)	
	Primary care	13 (1.8)		10 (1.5)		3 (5.3)		5 (1.2)			4 (1.7)		3 (6)		1 (16.7)	
	Other	88 (12.2)		82 (12.3)		6 (10.5)		66 (15.2)			13 (5.6)		9 (18)		0 (0)	
**Age of patients^e^**																
	Newborn to 12 years	160 (22.1)		152 (22.8)		8 (14)		114 (26.3)			41 (17.6)		5 (10)		0 (0)	
	13 to 17 years	171 (23.7)		157 (23.6)		14 (24.6)		103 (23.7)			64 (27.5)		4 (8)		0 (0)	
	18 to 54 years	405 (56)		368 (55.3)		37 (64.9)		265 (61.1)			118 (50.6)		18 (36)		4 (66.7)	
	55 to 69 years	364 (50.3)		329 (49.4)		35 (61.4)		231 (53.2)			109 (46.8)		20 (40)		4 (66.7)	
	≥70 years	294 (40.7)		262 (39.3)		32 (56.1)		173 (39.9)			96 (41.2)		21 (42)		4 (66.7)	
	All age groups	169 (23.4)		161 (24.2)		8 (14)		43 (9.9)			94 (40.3)		30 (60)		2 (33.3)	
	Other	13 (1.8)		13 (1.9)		0 (0)		9 (2.1)			3 (1.3)		1 (2)		0 (0)	

^a^OT: occupational therapist.

^b^PT: physical therapist.

^c^RT: respiratory therapist.

^d^DT: dietitian.

^e^Respondents could select ≥1 practice setting and ≥1 patient age group.

### Use of TR and Training Received

A summary of responses to items about TR-related training and use is provided in Table 2. In our sample, 19.5% (139/712) indicated that they had never used TR in their practice, and 8.8% (63/712) had been using TR before the COVID-19 pandemic (ie, >2 years). Half of the respondents (366/712, 51.4%) had been using TR for 1 to 2 years. PTs were late adopters and less likely to have used TR than OTs (*χ*^2^_1_=16.6; *P*<.001), and RTs were less likely than PTs and OTs (*χ*^2^_2_=87; *P*<.001). Overall, three-fourths (520/712, 73%) of all respondents (and 508/568, 89.4% of those currently using TR) indicated that their use of TR was specifically because of COVID-19; OTs were the most likely and RTs were the least likely to identify this as the reason (*χ*^2^_2_=70.9; *P*<.001). When those currently providing TR (553/712, 77.7%) were asked about continuing use of TR after the COVID-19 pandemic, 66.9% (370/553) indicated “yes,” 23.1% (128/553) indicated “maybe,” and 9.9% (55/553) indicated “no.” Across the 5 discrete age categories shown in Table 1, there was a gradual decline in the proportion of respondents using TR: children (145/158, 91.8%), youth (150/168, 89.3%), adults aged between 18 and 54 years (334/401, 83.3%), adults aged between 55 and 69 years (288/360, 80%), and adults aged >70 years (212/291, 72.9%).

Overall, respondents used TR for similar purposes, with most using it for teleconferencing (543/573, 94.8%) and teletreatment (478/573, 83.4%) and few for telemonitoring (137/573, 23.9%). Both video (546/573, 95.3%) and telephone (471/573, 82.2%) platforms were used frequently. Patients were most typically seen individually (549/573, 95.8%), but 23.7% (136/573) of the therapists used TR for groups. OTs were more likely than PTs to use TR for groups (104/379, 27.4% vs 23/173, 13.3%) and more commonly used video (371/379, 97.9% vs 159/173, 91.9%) and telephone (324/379, 85.5% vs 130/173, 75.1%) formats for TR delivery. Only 28.1% (203/723) of the respondents reported receiving specific training on TR delivery, with only 1.2% (9/723) indicating this to be part of their professional education (Table 2).

**Table 2:** Summary of telerehabilitation training and use responses.

Survey questions and response options			Total responses, n (%)	Site, n (%)			Profession^a^, n (%)		
				Canadian	Dutch	OT^b^		PT^c^	RT^d^
**Have you received any training in the provision of telerehabilitation services (or remote rehabilitation services)?^e^ (total responses: n=723; Canadian: n=666; Dutch: n=57; OT: n=434; PT: n=233; RT: n=50)**									
	Yes		203 (28.1)	196 (29.4)	7 (12.3)	120 (27.6)		79 (33.9)	4 (8)
	Part of my university professional training		9 (1.2)	9 (1.3)	0 (0)	5 (1.2)		4 (1.7)	0 (0)
	Professional continuing education offered at my place of work		125 (17.3)	122 (18.3)	3 (5.3)	76 (17.5)		45 (19.3)	4 (8)
	Professional continuing education offered other than my place of work		76 (10.5)	73 (10.9)	3 (5.3)	44 (10.1)		32 (13.7)	0 (0)
	Other		22 (3)	21 (3.2)	1 (1.8)	11 (2.5)		11 (4.7)	0 (0)
**How long have you been using telerehabilitation?** **(total responses: n=712; Canadian: n=657; Dutch: n=55; OT: n=430; PT: n=228; RT: n=48)**									
	I have never used telerehabilitation		139 (19.5)	129 (19.6)	10 (18.2)	51 (11.9)		55 (24.1)	32 (66.7)
	<6 months		61 (8.6)	54 (8.2)	7 (12.7)	37 (8.6)		21 (9.2)	2 (4.2)
	6 months to 1 year		83 (11.7)	78 (11.9)	5 (9.1)	38 (8.8)		38 (16.7)	4 (8.3)
	1 to 2 years		366 (51.4)	344 (52.4)	22 (40)	258 (60)		101 (44.3)	6 (12.5)
	2 to 5 years		47 (6.6)	37 (5.6)	10 (18.2)	36 (8.4)		10 (4.4)	1 (2.1)
	>5 years		16 (2.2)	15 (2.3)	1 (1.8)	10 (2.3)		3 (1.3)	3 (6.3)
**Are you using telerehabilitation due to the COVID-19 pandemic?** **(total responses: n=712; Canadian: n=657; Dutch: n=55; OT: n=430; PT: n=228; RT: n=48)**									
		Yes	520 (73)	484 (73.7)	36 (65.5)	350 (81.4)		153 (67.1)	13 (27.1)
**Which telerehabilitation services do you currently deliver or have delivered in the past (last 5 years)?** **(total responses: n=573; Canadian: n=528; Dutch: n=45; OT: n=379; PT: n=173; RT: n=16)**									
	Teleconsultation (video)		507 (88.5)	472 (89.4)	35 (77.8)	348 (91.8)		147 (84.9)	8 (50)
	Teleconsultation (phone)		432 (75.4)	396 (75)	36 (80)	294 (77.6)		121 (69.9)	12 (75)
	Teletreatment (video)		444 (77.5)	412 (78)	32 (71.1)	299 (78.9)		137 (79.2)	6 (37.5)
	Teletreatment (phone)		326 (56.9)	299 (56.6)	27 (60)	230 (60.7)		87 (50.3)	7 (43.8)
	Telemonitoring (video)		115 (20.1)	105 (19.9)	10 (22.2)	64 (16.9)		45 (26)	6 (37.5)
	Telemonitoring (phone)		108 (18.8)	95 (17.9)	13 (28.9)	66 (17.4)		33 (19.1)	8 (50)

^a^Dietitians are not included in the table owing to the small number of respondents (6/723, 0.8%).

^b^OT: occupational therapist.

^c^PT: physical therapist.

^d^RT: respiratory therapist.

^e^Respondents could select ≥1 response.

### Experience—Satisfaction and Confidence

A summary of respondents’ ratings on the 4 investigator-developed scales and the 2 standardized measures is provided in Table 3. Among all respondents (ie, those who did and those who did not provide TR services), many (197/712, 27.7%) reported having “some” experience with TR and being “moderately” confident with TR delivery. In follow-up with those providing TR, participants reported being “moderately to quite” satisfied with the care they provided and perceived it to be “moderately to quite” effective. Regarding the usability of the modes of TR that respondents had access to, the mean TUQ rating was 4.5 (SD 1.1) on a 7-point scale. The mTAM scores, indicating potential uptake of TR technology, were somewhat higher than usability, with a mean score of 4.9 (SD 1) on a 7-point scale. Among respondents who were currently using TR, the mean mTAM score was 5.01 (SD 0.92; 491/601, 81.7%), which was significantly higher than that of nonusers (mean 4.14, SD 1.1; t_141.9_=7.5; *P*<.001). There was no significant difference among professions on either the TUQ or mTAM measure (Table 3).

**Table 3:** Respondents’ mean (SD) ratings on perceptions of telerehabilitation use.

Rating scale	All responses, mean (SD)	Site, mean (SD)			Profession^a^, mean (SD)		
		Canadian	Dutch	OT^b^		PT^c^	RT^d^
Experience (n=712)	3.0 (1.2)	3.1 (1.2)	2.9 (1.2)	3.3 (1.2)		2.7 (1.1)	2.0 (1.2)
Confidence (n=712)	3.0 (1.1)	3.0 (1.1)	3.1 (1.2)	3.2 (1.1)		2.8 (1.2)	2.4 (1.2)
Effectiveness (n=553)	3.3 (0.9)	3.3 (0.8)	3.3 (0.9)	3.4 (0.9)		3.2 (0.9)	3.6 (0.8)
Satisfaction (n=553)	3.3 (0.9)	3.3 (0.9)	3.4 (1)	3.4 (0.9)		3.2 (0.9)	3.4 (0.9)
TUQ^e^—usability (n=524)	4.5 (1.1)	4.5 (1.1)	4.9 (0.9)	4.5 (1.1)		4.5 (1.1)	4.6 (1)
mTAM^f^—uptake (n=606)	4.9 (1)	4.8 (1)	5.3 (0.8)	4.9 (1)		4.8 (1.1)	4.9 (1)

^a^Dietitians are not included in the table owing to the small number of respondents (6/723, 0.8%).

^b^OT: occupational therapist.

^c^PT: physical therapist.

^d^RT: respiratory therapist.

^e^TUQ: Telehealth Usability Questionnaire; scored on a 7-point Likert scale: 1=disagree to 7=agree.

^f^mTAM: modified Technology Acceptance Model; scored on a 7-point Likert scale: 1=totally disagree to 7=totally agree.

### Barriers to and Facilitators of Using TR With Patients

Access to and confidence with technology were the most frequently selected facilitators of TR use. Among the 81 free-text responses in the “other” category, only 2 categories were mentioned by a minimum of 10 respondents: having an appropriate physical space (17/81, 21%) and access to appropriate technology for both provider and patient (10/81, 12%). Technology issues (463/520; 89%) and the need for physical contact (324/520, 62.3%) were the barriers selected by most respondents. Among the 101 “other” responses, 3 categories were reported by a minimum of 10 respondents: difficulty in observing movement or nonverbal responses (15/101, 14.9%), challenges with establishing a therapeutic relationship (10/101, 9.9%), and mismatch between patient’s characteristics and the available modalities (10/101, 9.9%; Table 4).

**Table 4:** Factors selected as facilitators of and barriers to telerehabilitation use.

Telerehabilitation factors and response options			All respondents (n=520), n (%)		Site, n (%)				Profession^a^, n (%)				
					Canadian (n=490, 94.2%)		Dutch (n=30, 5.8%)		OT^b^ (n=354, 68.1%)		PT^c^ (n=152, 29.2%)		RT^d^ (n=14, 2.7%)
**Which requirements are needed for you to be able to provide telerehabilitation?**													
	Patients’ electronic resources (e.g., access to internet, devices)	442 (85)		420 (85.7)		22 (73.3)		294 (83.1)		134 (88.2)		14 (100)	
	Good technology self-efficacy	395 (75.9)		383 (78.2)		12 (40)		284 (80.2)		97 (63.8)		14 (100)	
	Technology setup support	319 (61.3)		298 (60.8)		21 (70)		211 (59.6)		99 (65.1)		9 (64.3)	
	Educational material about the issue or condition	192 (36.9)		183 (37.3)		9 (30)		120 (33.9)		62 (40.8)		10 (71.4)	
	Use of online written information, or booklets	186 (35.8)		180 (36.7)		6 (20)		124 (35)		54 (35.5)		8 (57.1)	
	Good fit within workflow	183 (35.2)		167 (34.1)		16 (53.3)		118 (33.3)		62 (40.8)		3 (21.4)	
	Apps for a smart phone or tablet	167 (32.1)		157 (32)		10 (33.3)		114 (32.2)		48 (31.6)		5 (35.7)	
	Videos	146 (28.1)		137 (27.9)		9 (30)		98 (27.7)		42 (27.6)		6 (42.9)	
	Patient must have a chronic condition	14 (2.7)		14 (2.9)		0 (0)		5 (1.4)		3 (1.9)		6 (42.9)	
	I don’t know	10 (1.9)		10 (2)		0 (0)		8 (2.3)		2 (1.3)		0 (0)	
	Other	81 (15.6)		72 (14.7)		9 (30)		55 (15.5)		25 (16.4)		1 (7.1)	
**What barriers have you experienced delivering telerehabilitation?**													
	Technology issues (therapist or patient)	463 (89)		429 (87.6)		24 (80)		319 (90.1)		133 (87.5)		11 (78.6)	
	Lack of physical touch required to deliver services	324 (62.3)		308 (62.9)		16 (53.3)		211 (59.6)		105 (69.1)		8 (57.1)	
	Poor technology self-efficacy	194 (37.3)		190 (38.8)		4 (13.3)		136 (38.4)		53 (34.9)		5 (35.7)	
	Safety concerns	143 (27.5)		138 (28.2)		5 (16.7)		97 (27.4)		44 (28.9)		2 (14.3)	
	Privacy	116 (22.3)		106 (21.6)		10 (33.3)		91 (25.7)		23 (15.1)		2 (14.3)	
	Lack of appropriate training opportunities for therapists	115 (22.1)		110 (22.4)		5 (16.7)		82 (23.2)		31 (20.4)		2 (14.3)	
	Patients with acute conditions	99 (19)		94 (19.2)		5 (16.7)		61 (17.2)		32 (21.1)		6 (42.9)	
	Online platforms not designed for telerehabilitation	97 (18.7)		92 (18.8)		5 (16.7)		66 (18.6)		26 (17.1)		5 (35.7)	
	Poor fit within workflow as therapist	94 (18.1)		85 (17.3)		9 (30)		52 (14.7)		41 (26.9)		1 (7.1)	
	Lack of reimbursement by insurer for appropriate technology	70 (13.5)		59 (12)		11 (36.7)		42 (11.9)		26 (17.1)		2 (14.3)	
	Regulatory body policies	42 (8.1)		37 (7.6)		5 (16.7)		25 (7.1)		16 (10.5)		1 (7.1)	
	Inability to consult/collaborate with other professionals	31 (5.9)		30 (6.1)		1 (3.3)		24 (6.8)		6 (3.9)		1 (7.1)	
	I don’t know	7 (1.3)		7 (1.4)		0 (0)		4 (1.1)		3 (1.9)		0 (0)	
	None	1 (0.2)		0 (0)		1 (3.3)		1 (0.3)		0 (0)		0 (0)	
	Other	101 (19.4)		91 (18.6)		10 (33.3)		79 (22.3)		21 (13.8)		1 (7.1)	

^a^Dietitians are not included in the table owing to the small number of respondents (6/723, 0.8%).

^b^OT: occupational therapist.

^c^PT: physical therapist.

^d^RT: respiratory therapist.

## Discussion

### Principal Findings

This study aimed to obtain a descriptive overview of current TR practice among OTs, PTs, and RTs in Canada and the Netherlands and identify perceived barriers to and facilitators of practice. Most of our respondents (565/723, 78.1%) were OTs and PTs, with several years of clinical experience, working in primary care settings. Most respondents (366/712, 51.4%) had provided TR for approximately 1 to 2 years. Despite barriers such as technology issues and the limitations of not being able to provide hands-on care, 90.1% (498/553) of the respondents indicated that they were likely to continue to offer TR. This finding, in combination with emerging evidence suggesting that TR can be as effective as face-to-face care [31], points to the importance of continuing to attend to the needs of providers and consumers regarding ensuring effective TR delivery, beyond the COVID-19 pandemic.

In our survey findings, the application of TR was more frequent among OTs and PTs, compared with that among RTs. This finding may be more related to the practice areas of the RTs who responded to the study than a reflection of professional inclination toward TR use [32]. For example, most RTs (34/50, 68%) who responded worked in a hospital setting, whereas a high percentage of OTs and PTs who responded worked in private practice. A study by Almojaibel et al [33] surveying practitioners who provide pulmonary rehabilitation (primarily RTs) found that 79% of respondents had the intention of using TR to deliver pulmonary rehabilitation, with perceived usefulness, such as improving access for those in geographically remote locations, being the variable that most predicted planned use. Although this study did not specifically indicate the type of pulmonary rehabilitation setting, it is typically delivered via outpatient programs, suggesting that the practice setting rather than the profession may be a factor influencing therapists’ acceptance and uptake of TR.

In both Canada and the Netherlands, the COVID-19 pandemic drove a change in how rehabilitation services were delivered. Although the specifics of how each country has approached this change varied depending on the specific health care system and infrastructure in place and the severity of the COVID-19 outbreak in each country, it did not seem to influence the process of practice. For example, the use of TR remained quite close to traditional clinical practice such as conducting an intake or intervention via videoconferencing. Telemonitoring was less frequently used, especially among OTs, and this may be related to therapist-level factors, such as a lack of knowledge about or familiarity with the potential benefits of telemonitoring, or system-level factors, such as a lack of use of or support for this type of technology. Telemonitoring is not yet used to its full potential, and this mode of TR—and other options that are not investigated in this study—could become an integral part of rehabilitation interventions [34].

Despite limited training and equivocal self-efficacy for TR delivery, respondents who were providing TR were moderately to quite satisfied with their delivery, and 90.1% (498/553) of them indicated a desire to continue using TR in their daily clinical practice. Overall ratings of TR usability were moderate, suggesting that therapists felt competent to use the technology as intended. This is interesting considering that only 28.1% (203/723) of all respondents received any type of training related to TR delivery, most of which was “on the fly” rather than being part of their entry-to-practice education. Post hoc analysis (not reported in the *Results* section) indicated that more recent graduates were not more likely to have received training or to identify such training as having been obtained during their university program. Thus, there is no way to know if the therapists’ reports of being satisfied with TR delivery represent quality care through TR, as reported in recently published TR competencies, such as Health Information Technology Competencies [35]. This document identifies competence as baseline to expert skill level across 5 domains: direct patient care; administration; informatics; engineering, information systems, and ICT; and research and biomedicine. If TR is to become an integral part of rehabilitation practice, the curricula of OT, PT, and RT programs need to address TR competencies. A recent scoping review explored existing digital health competency frameworks for health care workers and provided recommendations for future digital health training initiatives and framework development [36]. They suggest that telehealth training initiatives should focus on competencies relevant to a particular health care profession, role, level of seniority, and practice setting. For rehabilitation professions, this could include skills such as functional strength assessments through observation only and enhancing communication tools such as motivational interviewing.

Therapists were increasingly less likely to use TR with older patients. This could be related to the level of acceptability of TR among older adults, as they have been found to be less likely than other age groups to choose TR [37]. However, the attitude of the therapist is also a factor in TR delivery, which leaves the question of whether agism is a factor in choosing a service delivery mode for older adults [38]. Respondents identified the need to ensure access, not just to the technology, but the right or appropriate technology that supports the needs of rehabilitation. Technical support for both health care provider and service recipient can create a smooth, more seamless delivery. In addition, TR modalities should be designed in an accessible manner so that they are easy to understand and use by people with impaired (digital) literacy, be available in several languages, and include different interfaces that are adjusted to user needs (eg, spoken language and pictograms instead of texts).

In terms of what facilitated TR use, it was primarily about the access and implementation of technology—ensuring that both recipient and provider of TR had access to the equipment required (ie, devices and internet access and bandwidth), there was technical support to set up the technology, and the provider felt confident in their TR delivery. To a lesser extent, having access to electronic resources relevant to their patient’s needs (eg, educational materials, websites, videos, and appropriate apps) was seen as an important facilitator. We may speculate that the therapists responding to this survey were seeking both the technology infrastructure and the skills and comfort in using this technology to reduce the multitasking demands of TR delivery so that they could focus on the *rehabilitation* component rather than the *tele* component. These findings highlight the context-specific experience of TR delivery among therapists in Canada and the Netherlands. As identified in the World Health Organization [2] recommendations document, TR benefit is dependent upon the specific health domain being addressed; development and evolution of interventions specific to that domain; available technology specific to these interventions; and a national infrastructure to support TR delivery including strategic prioritization, implementation and compliance policies and sufficient human resources and training to ensure equitable access to quality services.

The barriers that were identified through our survey echo findings in other studies of TR, indicating that these have yet to be adequately addressed. These barriers include concerns regarding patient safety, lack of technical support, loss of physical contact needed to conduct assessments, and more difficulty in developing rapport with the patient [39-41]. The loss of physical contact was of particular concern for PT respondents, corroborating the literature linking concerns related to remote contact impeding on safe monitoring of patients [42-44]. The lack of physical contact is an important area for further exploration, as best practice guidelines, while emphasizing the need for enhanced web-based intervention (such as improved education and advice), indicate that a hands-on physical assessment is key in musculoskeletal pain care [45]. However, so far, practice guidelines have not considered the mode of intervention delivery (ie, in person vs telehealth). Studies are needed to support decision-making among therapists regarding the type of therapy delivery that should be used for different diagnostic or functional issues and the most appropriate therapy delivery for different phases of the rehabilitation process. Furthermore, telemonitoring should be explored more as a potential tool to support safety monitoring during the initial PT assessment.

### Limitations

To the best of our knowledge, this is the first study to provide insight on TR uptake by multiple rehabilitation professionals during and after the COVID-19 pandemic. These insights contribute to further development of strategic planning for rehabilitation service delivery after the pandemic and addressing education needs related to TR competencies in professional preparation and educational programs. We were able to recruit many study participants from 2 different international contexts. However, the response rate was considerably high in Canada. The limited response from Dutch therapists can likely be attributed to our recruitment methods and the timing of the recruitment period. Despite this imbalance, the responses were generally quite comparable between the 2 countries, suggesting similar perspectives among therapists. Caution should be exercised in generalizing the study findings beyond the Canadian and Dutch contexts. For example, in the Netherlands, physiotherapists are regulated nationally, allowing them to practice TR across the country. In contrast, Canadian physiotherapists are regulated provincially. This structure requires physiotherapists to provide services only to individuals residing in their own jurisdiction. These jurisdictional boundaries may have influenced the responses by Canadian physiotherapists. Furthermore, given the low response rate, results from the Netherlands should be interpreted cautiously. Although the completion rate was quite high (606/723, 83.8%), we experienced some response attrition, which may have affected the reliability of questions further along in the survey. As with any voluntary survey, there is potential for response bias among therapists who chose to participate, such as private versus public practice, and responses may not be reflective of all practicing rehabilitation therapists. However, the relatively large sample size that included both TR users and nonusers provides us with great confidence in the validity of our findings. Low response rates from ETs and DTs precluded their inclusion in the analyses. Furthermore, conclusions about RTs’ perspectives should be approached with caution owing to the low response rate and the small proportion of therapists incorporating TR into their practice.

### Conclusions

In conclusion, to the best of our knowledge, this was the first study investigating rehabilitation professionals’ insight on TR uptake during and after the COVID-19 pandemic. TR practice was widely adopted in Canada and the Netherlands because of the COVID-19 pandemic, and most rehabilitation therapists (498/553, 90.1%) anticipate continuing to use TR in the future. Despite successful adaptation to this approach, rehabilitation therapists generally felt unprepared for TR delivery, and support for this transition was limited. Access to technology and confidence and competency with technology use were central barriers. Given the expectation that future practice will entail some combination of in-person and web-based delivery, great emphasis needs to be placed on enhancing TR competency through entry-to-practice education and continuing professional education.

## Abbreviations

DT: dietitian
ET: exercise therapist
ICT: information and communications technology
mTAM: modified Technology Acceptance Model
OT: occupational therapist
PT: physical therapist
RT: respiratory therapist
TR: telerehabilitation
TUQ: Telehealth Usability Questionnaire
